# A combined trade-off strategy of battery degradation, charge retention, and driveability for electric vehicles

**DOI:** 10.1038/s41598-024-71711-w

**Published:** 2024-09-23

**Authors:** Mohammed I. Tawfik, Ahmed Ali, Mostafa Asfoor

**Affiliations:** 1 Automotive Engineering Department, Military Technical College, I. Fangari, Cairo, 11766 Cairo Egypt; 2 Department of Vehicles, Ministry of Defense, Cairo, 11766 Cairo Egypt

**Keywords:** Battery aging, Electric vehicles, Genetic algorithms, Synthetic driving cycle, Optimal control, Vehicle modeling, Electrical and electronic engineering, Mechanical engineering

## Abstract

Electric vehicles are considered as an emerging solution to mitigate the environmental footprint of transportation sector. Therefore, researchers and automotive developers devote significant efforts to enhance the performance of electric vehicles to promote broader adoption of such technology. One of the critical challenges of the electric vehicle is limited battery lifetime and entailed range anxiety. In his context, development of counter-aging control strategies based on precise battery modeling is regarded as an emerging approach that has a significant potential to address battery degradation challenges. This paper presents a combined trade-off strategy to minimize battery degradation while maintaining acceptable driving performance and charge retention in electric vehicles. A battery aging model has been developed and integrated into a full vehicle model. An optimal control problem has been formulated to tackle the afore-mentioned challenges. Non-dominant sorting genetic algorithms have been implemented to yield the optimal solution through the Pareto-front of three contending objectives, based upon which an online simulation has been conducted considering three standard driving cycles. The results reveal the ability of the proposed strategy to prolong the life cycle of the battery and extend the driving range by 25 % and 8 % respectively with minimal influence of 0.6 % on the driveability.

## Introduction

### Background and motivation

Electric vehicles (EVs) are widely considered a promising alternative to traditional fossil fuel-based vehicles due to their ability to decrease emitted greenhouse gases (GHG) and reduce the dependency on fossil fuels. Therefore, EVs have attracted more and more attention in recent years due to their performance as zero-emissions vehicles, in addition to their superior energy- efficiency and performance. In spite of the such numerous advantages with EVs and many promotion efforts done by governments and legislation authorities, the market share in terms of overall sales remains rather limited^1^.

In addition to these considerations, scientists and automotive developers have a responsibility to enhance the competitiveness of electric vehicles compared to internal combustion engine vehicles (ICEVs) in various markets. In this context, the fundamental challenge for implementing electric vehicles is the battery, as it is expected to meet extensive requirements. The electric battery is the sole propulsion source for battery electric vehicle (BEV) and one of the two propulsion sources of hybrid electric vehicles (HEVs). Thus, batteries are required to provide power consistently and achieve sufficient energy capacity and density^2^.

The battery pack of a BEVs represents a significant portion of the overall vehicle cost; ranging from 25 to 30 %^3^. Regrettably, the battery degrades and loses capacity with time and usage, which mitigates its overall stored capacity, available power, and energy. Therefore, the major barrier to the large-scale adoption of EVs is the battery aging. Battery aging significantly impacts the energy storage capacity, power output capabilities, and overall performance of EVs. It also has implications for the cost and lifespan of the EV. The aging phenomenon can be classified in two terms: capacity and power fade. Capacity fade refers to a decrease in the battery’s ability to hold a certain amount of energy; it is measured in amp-hours and represented as a percentage. One the other side, power fade, is a reduction in the battery’s ability to provide power^4^.

There are two expressions to define battery life: cycle life and calendar life^5^. Calendar life is the amount of time left until the battery reaches to its end of live (EoL) during storage. Cycle life is the number of cycles that a battery can go through during its working process before losing 20 % of its initial capacity^6^. There are many processes and mechanisms that contribute to battery degradation. The major degradation mechanisms are solid electrolyte interphase (SEI) formation, transition metal dissolution (TMD), positive electrode structural decomposition, and metallic lithium formation. These degradation mechanisms are dependent on cell chemistry and storage as well as cycling conditions such as temperature, change in the State of Charge ($\Delta$SoC), charge, and discharge current^7^.

Moreover, battery degradation rate also influenced by driving habits and frequency, road condition, drive distance, and vehicle load. Battery temperature is considered the most important variable affecting battery degradation. Extreme temperatures, whether high or low, accelerate degradation of the battery. Temperatures above or below 25 lead to an increase in the aging rate. The desired working temperature range for most batteries used in electrified vehicles is typically $\hbox {15-35}\,^{\circ }$C^8^. The life span of the battery can be extended by ensuring that the battery temperature remains below <30 $^{\circ }$C^9^.

On the other hand, Overcharge and excessive depletion can significantly shorten the lifespan of a battery. The phenomenon of thermal runaway can occur as a consequence of overcharging, whereby additional external energy is introduced directly into the battery. Furthermore, the overall capacity of the battery can be decreased because of irreversible structural changes in both its cathode and anode, which are caused by excessive depletion^10^. Moreover, battery aging is influenced not only by temperature and SoC but also by the cumulative charge transfer into and out of the battery and the current magnitude in relation to the battery size (C-rate). Higher C-rate has the additional effect of increasing the internal temperature and hence it is common to observe capacity decrease and rapid aging^11^.

Additionally, fast charging leads to the formation of lithium plating, which is considered one of the major degradation mechanisms on the anode^12^. Moreover, high charging current accelerates the transfer of lithium ions to the anode, particularly at high SoC and low temperatures. Consequently, the flow of lithium ions surpasses the electrode’s capacity for intercalation, resulting in an accumulation of lithium ions on the surface of the anode. This leads to the formation of metallic lithium, which accelerates battery degradation^13^.

All the previous variables have an effect on battery life and degradation rate in operation, storage conditions, and even in a dormant state when the battery does not produce power. The aging of the battery in storage conditions is significantly affected by temperature and SOC, as high temperatures and high storage SoC accelerate the loss of capacity^14^. On the other hand, several factors, such as high or low working temperatures, cycling at high depth of discharge (DoD) or SoC, and high charge or discharge rates, accelerate cycle life^15^. During the charging process, the battery will inevitably lose some ot its initial capacity; however, retaining suitable temperature, cut-off voltage, and current can easily reduce the aging rate^16^. Similarly, the aging rate significantly increases with driving status with a significant influence of driver’s behavior, road condition, ambient temperature, and operating range of SoC^17^.

There are several solutions to effectively address range anxiety in EVs. Fast DC charging is a common solution to reduce the time it takes to charge the battery and extend the driving range, especially when traveling on highways. Other potential solutions include increasing the number of charging stations, utilizing battery swapping techniques, and improving battery technology to address range anxiety and reduce charging times^18^. Researchers and developers face significant challenges in developing battery technology that extends driving range while also addressing lightweight and limited volumes. Furthermore, it is crucial to design energy management systems (EMS) and vehicle models that can forecast energy consumption. However, each different driving scenario and behavior requires varying energy consumption. An adequate EMS can regulate this energy usage and even extend the driving range^19^.

### Relevant works in literature

The limited battery life of electric vehicles can anxiously impede their further development and broad adoption. Therefore, ensuring the congruence between the battery’s lifespan and that of the electric vehicle is crucial for the efficacy of EVs^20,21^. In order to address this matter, it is important to formulate models and operational techniques that facilitate comprehension, monitoring, and regulation of the battery aging phenomenon^22^.

The two main forms of battery aging models are electrochemical–physically based models and empirical or semi-empirical models^23^. Physics-based models are relatively accurate, yet not often utilized due to their complexity and the difficulty to be integrated to battery management systems (BMSs)^24^. On the other hand, semi-empirical models are increasingly developed to generate equations and parameters that can be fitted to gathered experimental data through extensive aging tests^25^. Although the predictability of such models is inferior compared to electrochemical ones, they are well-suited for real-time applications, owing to their compatible computational requirements and the ease of integration to online BMSs^26,27^.

Shuangqi Li et al. proposed an approach that aims to minimize the degradation cost of electric batteries^28^. Whereas the aging cost is determined by developing a degradation model using datasets obtained from accelerated aging tests. Based on quantifying and mitigating the cost associated with battery aging, the proposed approach achieved a reduction of 26.3 % in costs when compared to the BMS. Tang et al. adopted principal optimum control for the BMS of HEVs, considering dual objectives to minimize fuel consumption and capacity fading of the battery^29^. Additionally, the strategy aims to maintain the battery’s SOC within acceptable boundaries. The numerical solution for this multi-objective optimum control problem is obtained by applying Pontryagin’s minimum principle (PMP). This particular strategy demonstrated considerable effectiveness for aggressive driving behaviors.

In Ref.^30^, the authors investigated the impacts of fuel economy, emissions, and driveability performance on achievable energy management decisions. An enhancement in vehicle performance and a reduction in the fuel efficiency evaluation index by 20.26% could be realized. In addition, based on good convergence and distribution of the obtained Pareto solutions, the authors concluded that the solution sets may be used as a guidance for the selection of feasible plans and provide directions for initial design. Bin Zhou et al. presented a new equivalent consumption minimization strategy (ECMS), which aims to minimize the cost function while taking into consideration the battery aging. The simulation results demonstrate that the proposed algorithm effectively enhances the decay rate of accumulated ampere-hour throughput (Ah-throughput) while experiencing minimal or little fuel efficiency drawbacks as compared to the conventional approaches^31^.

In Ref.^32^, an electro-thermal aging model has been implemented to investigate an optimal BMS during the charging process. The optimization problem was formulated considering two contradictive objectives, i.e., charging speed and battery temperature. Non-dominated sorting genetic algorithms (NSGA-II) have been implemented to tackle the optimal solution (multi-stage constant current) to find minimal usage costs including the time-of-use price. Soren Ebbesen et al. presented a methodology for BMS in a parallel HEV via an optimal control approach. The purpose of this method is bi-fold: first, to reduce fuel consumption while ensuring that the battery’s SoC remains within acceptable limits, and second, to minimize the degradation process of the battery. Nevertheless, this particular approach failed to accurately forecast the reduction in capacity during practical driving scenarios and neglected to account for the influence of battery SoC on the entire process^33^.

In Ref.^34^, the authors developed an empirical model for a battery degradation based on experimental data that have been interpolated, with the exception of the number of cycles. The study demonstrated that aging rate is mostly influenced by temperature and discharging rate. Moreover, it has been observed that highway driving conditions are relatively more adverse to battery degradation due to entailed high depletion rates on such trips. Millner proposed another aging model for Lithium Ion batteries, which is based on theoretical models pertaining to the propagation of internal micro cracks^9^. The results indicated that it is crucial to avoid deep cycles over 60 % DoD, high temperatures exceeding <30 $^{\circ }$C, and high average SoC exceeding 60 % to ensure an maximal battery lifetime for EVs.

Two fast charging strategies for Li-ion batteries to minimize degradation by reducing the lithium plating have been proposed in Ref.^19^. The experimental results of the proposed strategies demonstrate that the battery life can be extended by 75 % and 250 % before reaching EoL, compared to the standard constant current – constant voltage (CC-CV) charging profile. Another five different SoC pre-conditioning strategies for vehicle-to-grid (V2G) applications have been presented in Ref.^35^. The study developed two semi-empirical degradation models to predict the calendar and cycling aging for batteries in EVs, tested using two driving profiles: aggressive and gentle. The results revealed that the proposed strategies can reduce the calendar and cycle aging combined up to 26.7 % for the first 100 days and varying to 12.3 % for one-year continual operation.

### Problem statement and main contribution

Based on the conducted review of recent works, the following gaps in the literature can be put forward: first, the inclusion of multiple objectives (3+ objectives) in cost function formulation calculation has been rarely considered. This aspect is crucial to be investigated since it puts forth the intra-dependency and contradiction between lifetime prolongation and other performance measures. Second, considering experimentally validated degradation models into the optimization process has been limited in literature. Besides, considering the driveability while implementing strategies to mitigate battery aging and reduce energy consumption has not been tested under various types of driving cycles. The latter aspect is significant to ensure the ability of any BMS to fulfill the driving requirements at different types of roads. Addressing the aforementioned gaps in research contributes to the ability to develop and achieve efficient vehicular control systems, that are capable of tackling undesired degradation of EV-batteries and ensure sufficient driving performance.

To this aim, this study proposes a multi-objective optimization strategy with two main goals: minimizing the battery degradation rate and maximize the vehicle’s driving range. Moreover, The proposed strategy takes into account the driveability performance of the vehicle over realistic driving cycles. A high-fidelity vehicle model has been implemented comprising longitudinal dynamics, battery performance and aging, and supervisory control module. A multi-objective optimization problem is formulated to minimize the capacity loss of the batteries and retain efficient energy consumption and driving performance. The formulated problem is solved using NSGA-II realizing an optimal trade-off solution that achieves balanced minimal values of considered objectives. In this study, the operating temperature is set between $23 ^{\circ }$C and $25 ^{\circ }$C and the operating range of SoC is set to 20 %–90 % to ensure a particular focus on the influence of driving cycles on battery aging.

The remainder of this paper is structured as follows: Sect. "Vehicle model" illustrates the modeling of vehicle dynamics and battery aging. validation of the two models is discussed as well. The description of the multi-objective optimization problem under consideration is provided in Sect. "Multi-objective optimization considering battery life prolongation". This section furthermore includes the proposed algorithm to solve the problem and the obtained Pareto front solutions. Finally, Sect. "Results analysis and discussion" addresses the conducting of a case simulation, accompanied by an analysis of the obtained simulation results. Conclusions are discussed in Sect. "Conclusion".

## Vehicle model

This section outlines the process of modeling and simulating the EV combined with a battery aging model. The mathematical model is divided from a conceptual point of view into vehicle longitudinal dynamics, electric drive-line, and battery aging model. The implemented model has been previously used and validated by the authors in multiple stages of this research^36–38^. However, we include further comparative analysis and verification of the model related to this work to enrich the discussion and comprehension of the proposed vehicle model. The objective of the modeling endeavor is to evaluate the battery aging under different driving scenarios and operating conditions by simulating it for various driving cycles^39^.

### Longitudinal dynamics

**Fig. 1:**
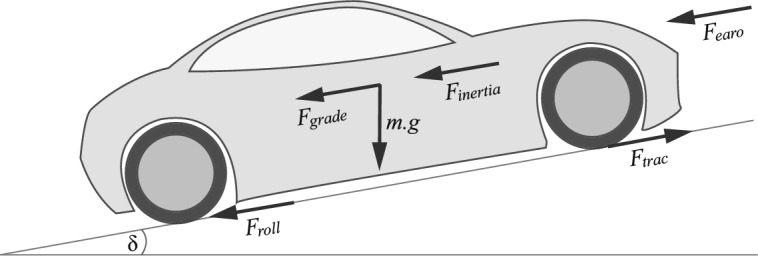
Free-body diagram of the vehicle exhibiting acting forces in longitudinal directions.

The fundamental goal of vehicle dynamics is to predict the performance of vehicles. Therefore, The vehicle model is split into subsidiary modules such as the driveline, the driver module, wheels, and tires. However, the study of vehicle dynamics relies not only on these modules but also on the interaction between them and the forces generated by the vehicle’s environment. It is essential to know the distribution of these forces that influence vehicle motion. The free-body diagram based on vehicle dynamics study of Reza N. Jazar is shown Fig. 1^40^.

The free-body diagram postulates he vehicle as a mass point, whereto the equations of motion can be formulated by equilibrium between the acting forces as

1 $$\begin{aligned} F_{trac}=F_{roll}+F_{earo}+F_{grade}+F_{inertia}, \end{aligned}$$

where $F_{trac}$ is the tractive force generated by the powertrain, $F_{roll}$ is the rolling resistance force resulting from the friction between the wheels and the road, $F_{earo}$ is the aerodynamic resistance generated by the airflow over the exterior of the vehicle body, and $F_{grade}$ is the force that appears in the case of a sloping road^41^. The inertial resistance $F_{inertia}$ is generated in the opposite direction to the motion during the acceleration and deceleration of the vehicle and can be formulated as:

2 $$\begin{aligned} F_{inertia}=M\frac{\partial }{\partial {t}}v, \end{aligned}$$

where *M* is a vehicle mass, and longitudinal velocity of the vehicle is $v_{veh}$.

It should mentioned that the longitudinal model is concerned with vehicle performance in longitudinal direction only (acceleration and braking) within the general coordinate system of vehicles^42^. The interaction with lateral and vertical dynamics is normally omitted due to its minimal impact on the vehicle in strain line motion^40,43^. According to Newton’s second law, Eq. (1) can be simplified as

3 $$\begin{aligned} M\frac{\partial }{\partial {t}}v=F_{trac}-F_{roll}-F_{earo}-F_{grade}, \end{aligned}$$

with the rolling resistance.

4 $$\begin{aligned} F_{roll}=C_{r}Mg\cos \delta , \end{aligned}$$

where *g* represents the gravitational acceleration, $\delta$ is the angle of road slope, and $C_{r}$ is the rolling resistance coefficient. The aerodynamic resistance is expressed as

5 $$\begin{aligned} F_{earo}= \frac{1}{2}\rho A C_{d} v_{veh}^2, \end{aligned}$$

where $\rho$ is the air density (1.25 $kg/m^3$ ), *A* is the vehicle projected frontal area, and $C_{d}$ is the aerodynamic drag coefficient. The grade force is expressed as

6 $$\begin{aligned} F_{grade}=Mg\sin \delta . \end{aligned}$$

### Electric drive-line

Calculation of the required traction torque ($T^{req}_{em}$) is carried out based on the difference between driving cycle speed and simulated vehicle speed as

7 $$\begin{aligned} T^{req}_{em}=k_{p}(v_{ref}-v_{veh}), \end{aligned}$$

where $k_{p}$ is the proportional gain^44^. The propulsion force is delivered by a permanent magnet synchronous motors (PMSM). The simplified mathematical model for the motor has been formulated using a single input/multiple output (SIMO) state space model as

8 $$\begin{aligned} \dot{x_{em}}&=A_\text {em}x_{em} + B_\text {em}v, \end{aligned}$$

9 $$\begin{aligned} y_{em}&= C_\text {em}x_{em} + D_\text {em}v, \end{aligned}$$

for

10 $$\begin{aligned} x_{em}&=\begin{bmatrix} \omega _{em} \quad i_\text {em} \end{bmatrix}^\text {T}, \ v=v_\text {em}(t),\end{aligned}$$

11 $$\begin{aligned} A_\text {em}&= \begin{bmatrix}-\frac{b_\text {c}}{J_{m}} &  \frac{k_\text {et}}{J_{m}}\\ \\ -\frac{k_\text {et}}{L_{e}} &  -\frac{R_\text {e}}{L_{e}} \end{bmatrix}, \ B_\text {em} = \begin{bmatrix} 0\\ \\ \frac{1}{L_{e}} \end{bmatrix},\end{aligned}$$

12 $$\begin{aligned} \ C_\text {em}&=\begin{bmatrix}1& 0\\ \\ 0& 1\end{bmatrix}, \ \text {and} \ D_\text {em} = \begin{bmatrix}0\end{bmatrix}, \end{aligned}$$

where $k_\text {et}$ denotes the electromotive torque constant, $J_{m}$ the rotational moment of inertia, $b_\text {c}$ the viscous friction constant, $R_\text {e}$ the equivalent circuit resistance, and $L_{e}$ the equivalent inductance. $i_{em}$ denotes motor current, $\omega _{em}$ is rotational speed, and $v_\text {m}$ applied motor voltage.

The electric machine can function both as a motor and a generator^45^. When it operates as a motor, it obtains energy from the battery to generate propulsion. On the other hand, when it operates as a generator, the battery gets charged through the electric machine, either in the charging mode or the regenerative braking mode^46^. The efficiency of the electric motor ($\eta$) depends on both the torque *T* and speed $\omega _{em}$ of the motor, which can be represented by a steady-state map. The required power output of the electric motor can be expressed as

13 $$\begin{aligned} P_{em}=T\cdot \omega _{em}\cdot \eta ^e(\omega _{em}, T_{em}), \end{aligned}$$

where the value of $e=-1$ when the electric machine acts as a motor, and $e=1$ in any other cases.

The slow variation of the SoC of the battery is a critical factor in power management^47^. Therefore, it is essential to develop a battery model that accurately represents the relationship between voltage, resistance, and SoC.

**Fig. 2:**
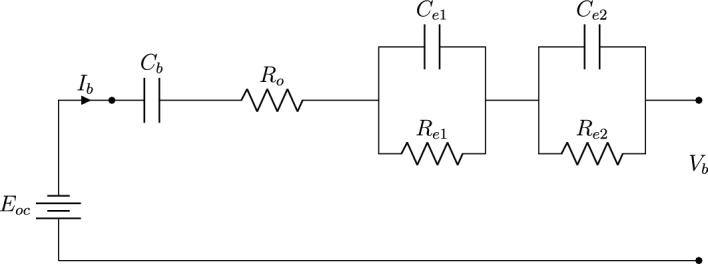
Equivalent circuit model of the battery based on second-order Thevenin model.

In this study, the battery pack is represented by a second-order Thevenin (PNGV)^37,38^. Thevenin model is an accepted approach for optimizing electric powertrains while maintaining the accuracy and reliability of individual cell dynamics^48^. Figure 2 illustrates the second-order Thevenin (PNGV) model including three elements: the open-circuit voltage ($E_{oc}$), internal resistances, and corresponding capacitances.

The internal resistances consist of three components: ohmic resistance ($R_{o}$), and equivalent Thevenin resistances ($R_{e1}$) and ($R_{e2}$). $C_{e1}$ and $C_{e2}$ denote the equivalent Thevenin capacitance for each RC-network^38^. $C_{b}$ is the fictive capacitance representing the changes in electromotive force and $i_b$ represent the battery charging/discharging current. Thus, the battery voltage $V_b$ is calculated as

14 $$\begin{aligned} V_\text {b}&= E_\text {oc} - i_\text {b} \ R_\text {o} - v_\text {e1} - v_\text {e2} - \frac{1}{C_\text {b}} \int _{t_\text {i}}^{t_\text {f}}i_\text {b}\ dt, \end{aligned}$$

where are $v_{e1}$ and $v_{e2}$ are voltages across RC network.

The polarization dynamics during battery charging and discharging are modelled through two series RC-Networks as^49^

15 $$\begin{aligned} \begin{bmatrix} \dot{v}_\text {e1} \\ \dot{v}_\text {e2} \end{bmatrix} = \begin{bmatrix} - \frac{1}{R_\text {e1} C_\text {e1}} &  0 \\ 0 &  - \frac{1}{R_\text {e2} C_\text {e2}} \end{bmatrix} \ \begin{bmatrix} {v}_\text {e1} \\ {v}_\text {e2} \end{bmatrix} + \ \begin{bmatrix} \frac{1}{C_\text {e1}} \\ \frac{1}{C_\text {e2}} \end{bmatrix} \ i_\text {b}. \end{aligned}$$

Hence, the power of the battery $P_{b}$ can be expressed as

16 $$\begin{aligned} P_{b}=V_{oc}I_{b}-R_{i}I_{b}^2, \end{aligned}$$

and battery SoC can be subsequently determined using the coulomb counting method, which involves calculating the SoC based on the battery current as

17 $$\begin{aligned} SoC(t)=SoC_{0}-\frac{1}{Q_{b}}\int _{t_{0}}^{t_{t}}I_{b}(\tau )\ d\tau , \end{aligned}$$

where $Q_{b}$ is the battery capacity. The output variables of the battery model is directly connected to the inputs of the electric model motor such that $V_b \equiv v_{em}$ and $i_b \equiv i_{em}$. The parameters of vehicle dynamics, drive-line specs, electric motor, and battery model are listed in Table 1. Vehicle speed and acceleration, tractive force, and aerodynamic resistance are determined and updated at each time step.

**Table 1:** Specifications of electric vehicle components and key parameters.

Parameters	Value
Gross vehicle weight	1620 [Kg]
Related motor torque	250 [Nm]
Related motor power	125 [kW]
Battery capacity	18.8 [kWh]
Vehicle frontal area (A)	2.38 [$\hbox {m}^2$]
Aerodynamic drag coefficient (*Cd*)	0.29
Rolling resistance coefficient ($C_{roll}$)	0.02
Gravity (g)	9.81
Road angle	0

### Battery aging model

In this work, a semi-empirical model is applied to the battery to evaluate the capacity loss. The model is based on damage accumulation, which utilizes the concept of accumulated charge throughput by establishing a connection between EoL and Ah-throughput. The underlying concept is that a battery, when subjected to various operational parameters such as temperature $\theta$, state of charge *SoC*, and C-rate $I_{c}$, can attain a cumulative throughput before reaching its EoL^50^.

This model employs the normalized capacity loss, denoted as $Q_{loss}$[%], as an indicator for evaluating battery degradation as

18 $$\begin{aligned} Q_{loss}(I_{c},\theta ,SoC,Ah)=\sigma _{funct}(I_{c},\theta ,SoC) \cdot Ah^z, \end{aligned}$$

where *Ah* is the accumulated charge throughput, which refers to the total amount of charge that is capable of flowing into and out of the battery^51^. The power law exponent, denoted as *z*, represents the Ah throughput dependence and $I_{c}$ is defined as

19 $$\begin{aligned} I_{c}=\frac{|I_{batt} |}{Q_{batt}}. \end{aligned}$$

As for $\sigma _{funct}(I_{c},\theta ,SoC)$, it is a non-linear severity factor function that can be expressed as

20 $$\begin{aligned} \sigma _{funct}(I_{c},\theta ,SoC)=(\alpha \cdot SOC + \beta ) \cdot \exp {(\frac{-E_{a}+ \eta \cdot I_{c}}{R_{g} \cdot (273.15 + \theta )})}, \end{aligned}$$

where $E_{a}$ is the activation energy equal to 31,500 [$\hbox {Jmol}^{-1}$]^52^, $R_{g}$ is the universal gas constant, $\eta$ is the $I_{c}$ dependence, $\alpha$ and $\beta$ define *SoC* dependence. To identify the model parameters in Eq. (20), Onori et al. conducted an approach based on nonlinear least squares is to evaluate the experimental data obtained from^52^ for three profiles of lithium iron phosphate batteries (LiFePO$_{4}$). These batteries were subjected to several constant operating conditions, including temperature, C-rate, and SoC. The nonlinear identification toolbox from MATLAB is employed for the objective of identifying the model parameters through minimization of the overall error^51^.

The values of *z* for the three profiles show a high degree of proximity. Therefore, the average value of *z* (=0.57) is selected as the basis for further identification of the aging model. The optimal values of variables $\alpha$ and $\beta$ are shown in Table 2. The most suitable value of $\eta$, which yields the best fit, is determined to be 152.5.

**Table 2:** Optimal values of $\alpha$ and $\beta$.

Parameter	*SoC* [%] $\ge$ 45	*SoC* [%] < 45
$\alpha$	2694.5	2896.6
$\beta$	6022.2	7411.2

The battery aging model is developed to evaluate the degradation of a battery considering the three key-parameters that influence degradation rate, namely: SoC, C-rate, and temperature, which can be expressed as

21 $$\begin{aligned} Q_{loss,\%}=(\alpha \cdot SoC + \beta ) \cdot \exp {(\frac{-E_{a}+ \eta \cdot I_{c}}{R_{g} \cdot (273.15 + \theta )})} \cdot Ah^z. \end{aligned}$$

**Fig. 3:**
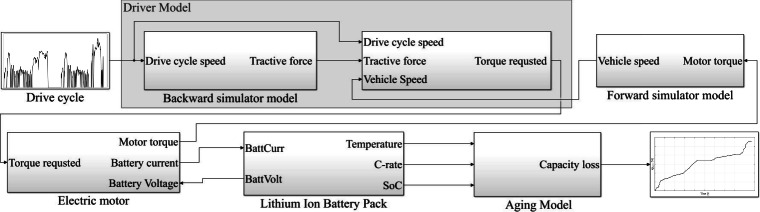
Electric vehicle and battery aging Simulink model.

By combining the battery aging model with the EV model as a comprehensive vehicle simulator, it becomes more possible to understand how a battery or a pack of several batteries would behave in a specific driving scenario or under various driving conditions as illustrated in Fig. 3.

### Model validation and simulation

In order to validate the accuracy of the simulated EV model, the simulation was conducted utilizing the present model and the results were compared with the chassis-dynamometer data obtained from Argonne National Laboratory (ANL) for the same vehicle^53^. To this aim, the simulation model is integrated as a software-in-the-loop to follow the dynamometer driving schedule, which comprises three distinct driving scenarios to reflect different conditions to which the vehicle is exposed.

**Fig. 4:**
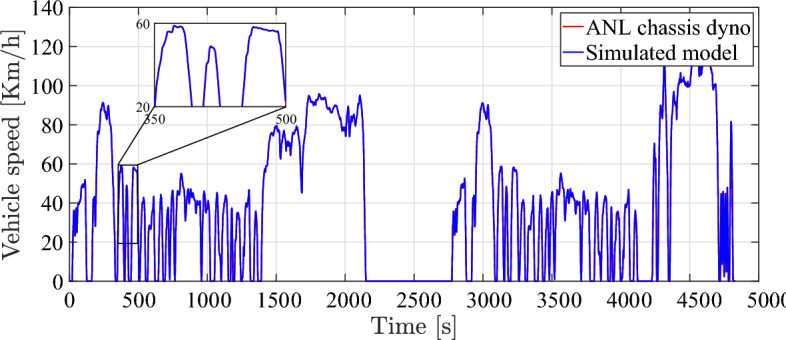
Vehicle speed over time during simulation.

**Fig. 5:**
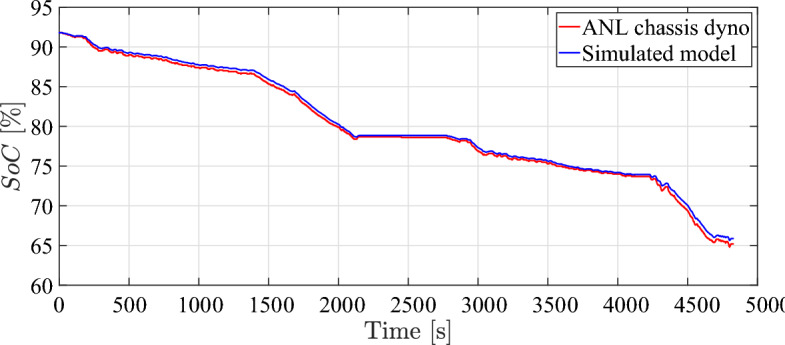
Battery SoC over time during simulation.

**Fig. 6:**
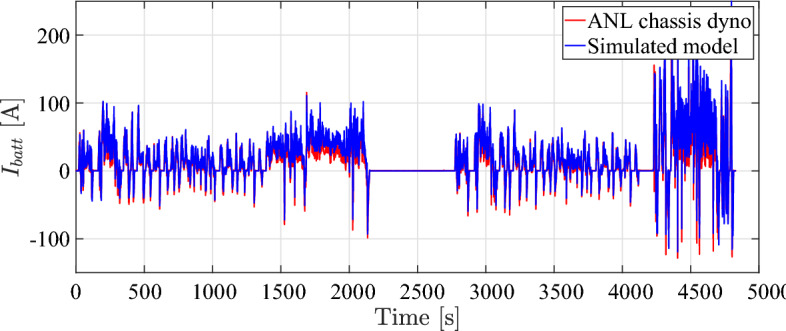
Battery output current over time during simulation.

**Fig. 7:**
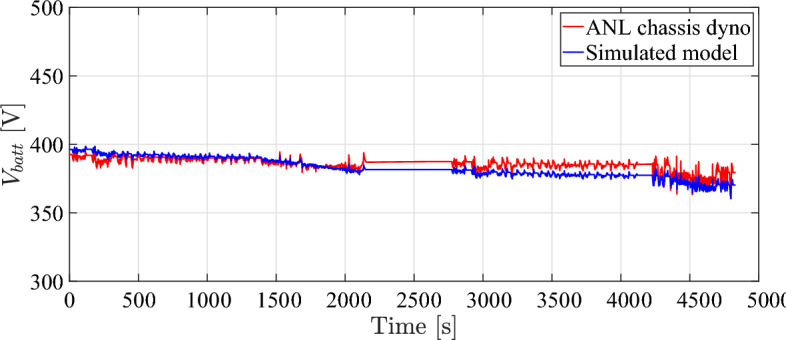
Battery output voltage over time during simulation.

EV model speed compared to dyno drive cycle speed is shown in Fig. 4. It can be observed that the simulated vehicle has the ability to precisely follow the drive cycle input with minimal error, while simultaneously satisfying the specified speed and torque response criteria. The plot illustrated in Figs. 5, 6, and 7 demonstrate a satisfactory level of concordance for battery performance over time between the simulated model and ANL measured values of battery SoC, battery current, and battery voltage, respectively.

The simulation results in comparison to ANL dataset and the root-mean-square error (RMSE) are reported in Table 3. The final RMSE value was, indicating a satisfactory level of agreement between the two sets of data. Moreover, the simulation accuracy for all simulation results is more than 95 %. This level of proximity between simulated results and experimental data validates fundamental assumptions used in the simulation model of the electric powertrain.

The battery capacity loss of the model (21) is also compared to the capacity loss data obtained from experimental data In order to verify the aging model, wherein both are subjected to identical operating conditions. The investigations on aging were conducted by Pierfrancesco Spagnol et al. within the battery characterization and aging laboratory located at the Center for Automotive Research of The Ohio State University^54^.

**Fig. 8:**
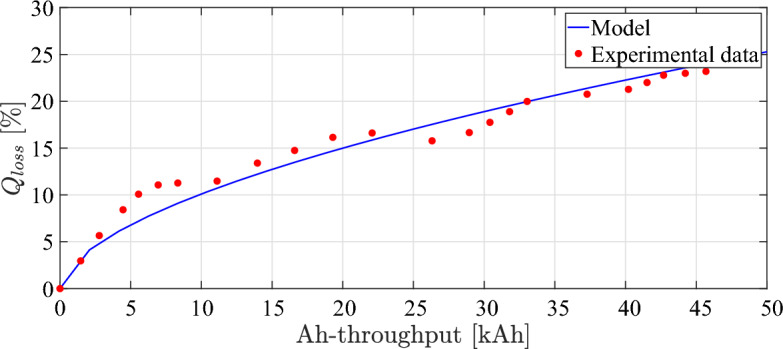
Curve fitting of identified aging model with the experimental data.

The battery cell being tested is LiFePO4 (ANR26650) from A123 systems, characterized by a nominal voltage of about 3.3V cell and a nominal capacity of 2.3 Ah. The specified operating conditions are defined in terms of the average $\overline{SoC} = 42$ [%], average C-rate $\overline{I_{c}} =3$ [1/h], and average battery temperature $\overline{\theta } =38$ [$^{\circ }$C]^55^. The results of capacity model that has been identified is closely correspond to experimental data points as illustrated in Fig. 8.

**Table 3:** Simulation results compared to ANL dyno test results.

Data	Value	Actual test	Simulated model	RMSE	Accuracy
Speed [km/h]	Max.	129.11	129.17	0.80	99.69 [%]
	Min.	0.0	0.0		
State of charge [%]	Max.	91.8	91.8	0.38	99.66 [%]
	Min.	64.8	65.7		
Current [A]	Max.	239	250	11.5	95.2 [%]
	Min.	– 128.5	– 120		
Voltage [V]	Max.	394.5	398.5	5.2	98.7 [%]
	Min.	360.7	360.3		
Distance [km]		53.27	53.27	0	100 [%]

## Multi-objective optimization considering battery life prolongation

In this work, a multi-objective optimization problem is formulated to address battery degradation and limited the driving range of the studied EV under a specified driving conditions. In this formulation, selected design variables are set to indicate the relationship between the battery SoC, capacity loss of the battery, and the powertrain performance. Hence, the objectives of optimizing vehicle energy consumption and the output power of the powertrain to sustain the driving performance usually conflict with each other. Therefore, the optimization procedures are developed to tune the design variables, that mainly affect the drop in battery SoC and degradation rate while considering powertrain output power required for vehicle propulsion.

Multi-objective genetic algorithms (MOGAs) is a category of computational tools that utilize genetic algorithms (GAs) to address complex optimization problems characterized by multiple conflicting objectives. It can provide numerous sets of solutions, which adhere to the Pareto principle and are commonly referred to as the Pareto solution set. GAs operate by manipulating a population of feasible solutions, utilizing the principle of “survival of the fittest” to produce enhanced approximations to a specified solution. In this regard, NSGA-II, proved a particular competence to handle nonlinear and conflicting objectives, explore feasible spaces for design variable, and fulfill complex constrains efficiently at reduced computational requirements^37^. Hence, NSGA-II has been considered to solve the multi-objective optimization problem in this work.

### Mathematical formulation

The multi-objective optimization problem aims to address three objectives concurrently: first, battery capacity loss; second, charge retention; and third, the disparity between power delivered from the battery and required tractive power to propel the vehicle. A mathematical formulation for the multi-objective problem of the electric driveline can be formulated as

22 $$\begin{aligned} \mathrm {{\textbf {J}}}(x,t) = \begin{bmatrix} J_1(x,t) \ \ \ J_2(x,t) \ \ \ J_3(x,t) \end{bmatrix}, \end{aligned}$$

for

23 $$\begin{aligned} J_1(x,t)&= Q_{loss}(x,t), \end{aligned}$$

24 $$\begin{aligned} J_2(x,t)&= \Delta SoC(x,t), and \end{aligned}$$

25 $$\begin{aligned} J_3(x,t)&= \Psi (x,t), \end{aligned}$$

where $\Psi$ denotes the vehicle driveability, which is calculated through the RMSE between the tractive power and battery power as

26 $$\begin{aligned} \Psi = \sqrt{\frac{1}{N}\sum _{i=1}^{N}{({P_i}^{trac} -{P_i}^{batt})^2}}, \end{aligned}$$

where *N* represents the number of the measured points, ${P_i}^{trac}$ and ${P_i}^{batt}$ represent the tractive power and battery power at point *i*, respectively^56^.

Selecting an appropriate weighting factor can be challenging because the three objectives have different scales and are simultaneously considered in the problem. Therefore, the objectives $J_1, J_2,$ and $J_3$ have been normalized without any weighting factors for each objective. This step has been conducted due to the proved benefits of normalization in multi-objective optimization and to avoid prioritization of any particular objective, which might mislead the interpretation of the results^57,58^. Furthermore, considering a specific weighting factors during the optimization to achieve some case-relevant results (i.e. prioritizing driveability or battery lifetime) is an on-going extension of our research, yet not presented in this manuscript.

If the objective is to optimize all parameters, the optimization process might become quite complex, resulting in a substantial computational burden and prolonged computing time. Furthermore, it is possible that it may not provide an optimal solution^36^. Therefore, the authors select one of the powertrain system’s parameters as a variable for optimization, which significantly influences the optimization objectives. These parameters refer to the battery current and the change of the current over time. The current extracted from the powertrain depends mainly on the power required to propel the vehicle and spontaneously affects the battery degradation rate. Therefore, the control variables in *x* can be described as

27 $$\begin{aligned} x&= \begin{bmatrix} {i} \ \ \ \frac{\partial }{\partial {t}} {i} \end{bmatrix} , \end{aligned}$$

where *i* is battery current and $\frac{\partial }{\partial {t}} {i}$ is change of current over time. The control variables are subjected to constraints

28 $$\begin{aligned} {i}&\le {i}_{max}&and \end{aligned}$$

29 $$\begin{aligned} {\frac{\partial }{\partial {t}} i }&\le \frac{\partial }{\partial {t}}i_{max}. \end{aligned}$$

The depicted battery aging model in Eq. (21) gives an insight into the set of variable that influence the capacity loss rate of batteries, including the operating SOC, cell temperature, current flow dynamics, DOD, and the remaining throughput of the battery. In this contribution, a particular focus has been given to the current flow dynamics, represented in $[ {i} \ , \ \frac{\partial }{\partial {t}} {i} ]$, which proved a significant potential to mitigate battery degradation^37,38^. The operating temperature of the battery is another efficient variable to reduce battery degradation; however, it highly influences the required cooling power, which directly accelerates the depletion rate of the battery. Thus, deploying further influencing variables on capacity loss is considered as a short-term upgrade of our work.

### Solving algorithm

In this work, NSGA-II have been considered as the solving algorithm, which is well-recognized as an efficient solver for complex vehicular control problems^59^. The simplification of the NSGA-II results in several benefits, including enhanced computational efficiency and improved convergence of the solution set^60^. Additionally, it has the capability to extend solution sets to cover the entirety of the Pareto domain, guaranteeing both uniformity and diversity within the solution sets. The setting parameters of utilized NSGA-II are detailed in Table 4.

**Table 4:** NSGA-II algorithm parameters.

Parameters	Value
Population size	50
Maximum number of generations	100
Crossover rate	0.8
Mutation rate	0.1
Pareto fraction	0.2

The algorithm starts by generating a stochastic initial population. Subsequently, it utilizes the individuals from the current generation to create the next population. This is achieved by selecting individuals with the best fitness values as elite and choosing parents with high expectations followed by the subsequent production of offspring from these parents. The problem has been computed in the same MATLAB environment, which enabled further analysis of battery capacity loss, charge retention, and vehicle performance. Eventually the algorithm finds the best values of control variables to mitigate the battery degradation rate and drop of the SoC while maintaining the required power for vehicle propulsion. Then the minimum values for the three objectives are identified by defining the total cost function as the summation of equally-weighed objectives.

### Establishment of the case-relevant Pareto front

**Fig. 9:**
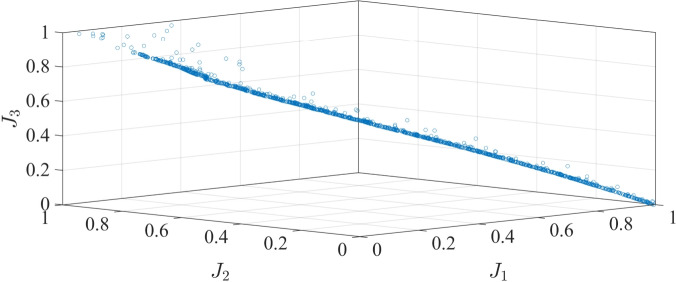
3-D Pareto front of normalized objectives $J_1$, $J_2$, and $J_3$.

The three-dimensional Pareto solution set for a multi-objective optimization problem is shown in Fig. 9. Each of the axes represents one of the three objective functions. The Pareto solution sets demonstrate good characteristics in terms of distribution and convergence, thus satisfying the standards of the multi-objective optimization norms. Furthermore, the relationships among driveability, battery capacity loss, and charge retention reveal achievable equilibrium properties. This indicates that the solution is set up to provide a broader range of design options for the EV model.

**Fig. 10:**
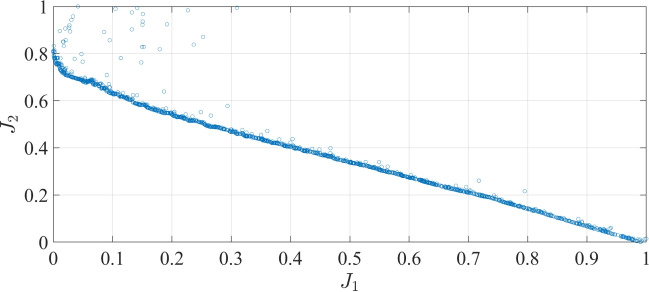
Pareto-front of $J_1$ and $J_2$.

**Fig. 11:**
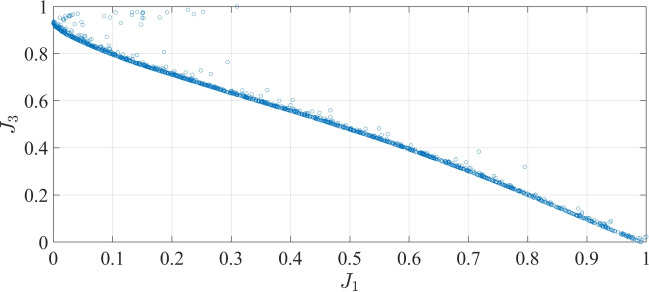
Pareto-front of $J_1$ and $J_3$.

In addition, the relationship between each two consequential objectives is illustrated in Figs. 10 and  11. It is obvious that the two objectives in each figure were contrasted. Hence, in cases when a single target is overly maximized, it will lead to a rapid deterioration of the other objective. Therefore, the optimal solution for the multi-objective optimization problem is selected, which demonstrates the minimum values for the three specified objectives. It is noticeable that the yielded optimal strategy is custom-optimized for a specific profile (set of driving cycles), which can be applied to similar fixed routes. Moreover, the attained offline solution can be implemented for online vehicular control as an on-board library.

The search space for the control variables ${i}_{max}$ and $\frac{\partial }{\partial {t}}i_{max}$ has been set to [80 350] [A]and [500 2000] [A/s] accordingly. The determination of space size is based on the average values of both variables during implemented driving cycles. The optimal solution has been obtained based on the minima of all objectives simultaneously on the Pareto front at ${i}_{max} = 110$ [A] and $\frac{\partial }{\partial {t}}i_{max} = 1000$ [A/s], which reflects the achieved results in the sequel.

## Results analysis and discussion

In this section, the models are integrated with the optimization algorithm and simulated under a specified set of driving cycles to verify the proposed optimization strategy. The specified driving scenario comprises three standard driving cycles, namely: urban dynamometer driving Schedule (UDDS), highway fuel economy driving Schedule (HWFET), and US06. The UDDS reflects in-city driving conditions, while the HWFET pertains to highway driving conditions below 96 km/h. On the other hand, US06 is a driving schedule characterized by high acceleration and aggressive behaviors. the total distance of the combined drive cycle is 53.27 km, which can be assumed as a one-day drive. The ambient temperature variation is considered to be between $23 ^{\circ }$C to $25 ^{\circ }$C.

**Fig. 12:**
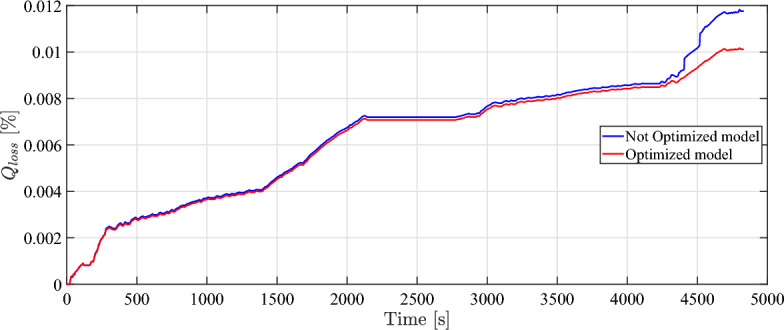
Comparison of $Q_{loss}$ in optimization mode to normal model over time.

For a one-day trip, Fig. 12 illustrates the degradation of the battery for the optimized and not optimized models. In the case of the unoptimized model, it can be observed that the rate of battery capacity loss significantly increases over the US06. This is assigned to the higher acceleration characteristics of the US06 cycle in comparison to the HWFET and UDDS cycles. The reason for this is that the US06 cycle imposes greater demands on the powertrain of the vehicle, resulting in a substantial discharge of charge from the battery and requiring a higher C-rate. As a result, this leads to an increase in battery capacity degradation.

However, in the case of the optimization strategy employed in the models, the curve experiences a decrease during the whole trip, especially during the US06 cycle. It concludes that the implemented optimiztion approach decreased the battery capacity loss by 15.8 % after only one-day trip that endured nearly 80 minutes over 53.27 km. Due to the nonlinearity of battery degradation rate, the percentage of mitigated capacity loss is anticipated increase over long periods of driving and operation.

**Fig. 13:**
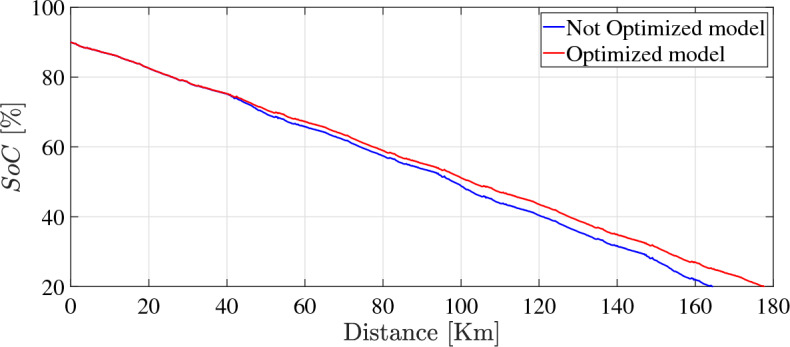
Comparison of *SoC* in optimization mode to normal model over distance traveled.

It should be pointed out clearly that above-mentioned results have been achieved for only one repetition of the driving scenario (including 3 standard driving cycles) for approximately 4800 seconds (1.3 hours). The absolute loss rate after such a short period is negligible using any control strategy. To investigate the achievable reduction in capacity loss, the model has been simulated considering a continuous repetition of the driving scenario on daily basis for nearly 2900 days until reaching the EoL of the battery as shown in Fig. 13.

**Fig. 14:**
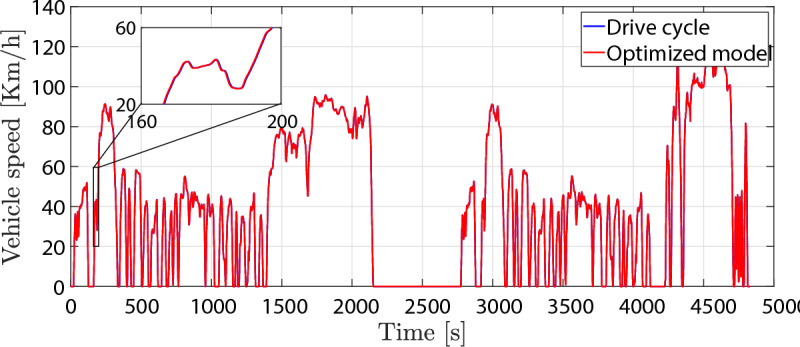
Comparison of vehicle speed in optimization mode to drive cycle speed over time.

It can be perceived from the illustrated results that the driving range has been extended by 8% owing to the proposed optimization strategy. Furthermore, the optimization strategy has been able to yield a reduction of energy consumption by 13 %. Additionally, the optimization approach has demonstrated satisfactory results regarding driveability performance, as shown in Fig. 14.

It can be seen from the figure that the vehicle speed in the optimized model follows the drive cycle speed very well. Given that the relative error percentage between the two speeds is 0.6%, it satisfies the objectives that have been established. The simulation results of the optimized model and conventional model are reported in Table 5.

**Table 5:** Optimization results compared to normal condition results.

Data	Normal model	Optimized model	Improvement [%]
Capacity loss ($Q_{loss}$) [%]	0.012	0.0101	15.8
Driving range [Km]	164.5	177.6	8
Energy consumption [WH/Km]	171.3	149.2	13

In order to identify the battery life cycle, the simulations are conducted based on the following assumptions: (1) the driving cycle remains the same for every trip; (2) one-day driving consists of two UUDS, one US06, and one HWFET, then it is replicated until the SoC reaches 20%; (3) The vehicle covers 53.27 Km per day; (4) the SoC window set between 90% and 20% to avoid over-charge and over-discharge; (5) driving conditions are the same for each day; (6) one year consists of 365 days; (7) The ambient temperature variation between $23 ^{\circ }$C and $25 ^{\circ }$C; (8) the simulation will run until the battery reaches to its EoL when it degrades to 80% of its initial capacity^61^.

**Fig. 15:**
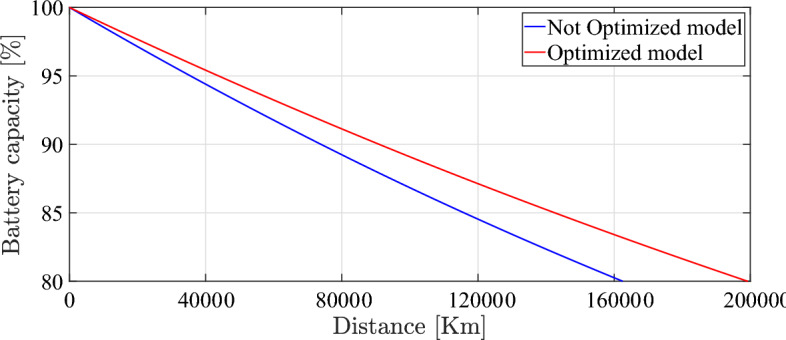
Battery degradation over distance traveled.

**Fig. 16:**
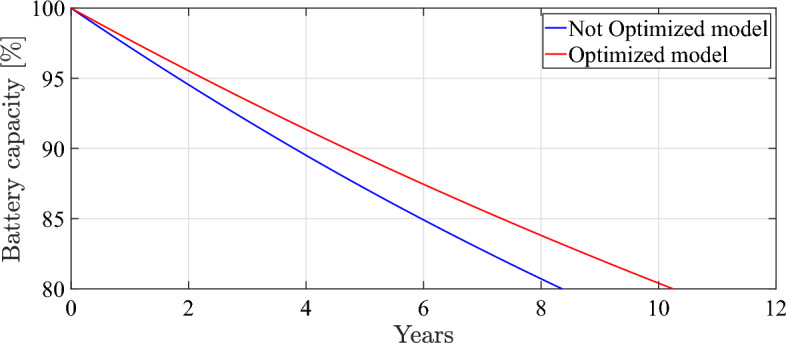
Battery degradation over years.

Figures 15 and  16 show the battery degradation curve form the initial capacity of the battery to reaching the EoL over distance and time respectively. It can be seen from Fig. 15 that, before optimization, the vehicle covers distances of 160,000 km, whereas, in optimized mode, the vehicle covers a distance of nearly 200,000 km.

Regarding Fig. 16, the battery reaches 80% of its initial capacity in about 8 years, whereas, in optimized mode, the battery reaches the EoL in about 10 years. To conclude, after simulated the optimized and unoptimized models to the EoL of the battery, it demonstrated the ability of the optimization strategy to extend the life cycle of the battery by 2 years (25%). The demonstrated results and the entailed improvements in capacity loss, energy consumption, and driveability has been entirely calculated based on numerical simulation for the entire time horizon until reaching the EoL of the battery without any extrapolation or anticipation.

## Conclusion

This paper presented a multi-objective optimization strategy for electric vehicles, based on simultaneous minimization of battery deterioration rate while retaining satisfactory driving performance and charge retention. A high-fidelity vehicle model has been implemented comprising a battery aging model to calculate the influence of driving behavior on the normalized capacity loss of the battery. A comparative analysis between simulated results and experimental dynamometer testing results has been conducted to verify the accuracy of both longitudinal dynamics and battery aging results. An optimal control problem has been formulated comprising three conflicting objectives, namely: capacity loss, on-board charge retention, and driveability. Non-dominant sorting genetic algorithms have been implemented to solve the optimization problem based on establishing the Pareto-front amongst objectives and yielding the optimal solution considering the normalized minima of all objectives.

For the testing procedures, three standard driving cycles (US06, HWFET, and UDDS) have been combined to form a daily driving scenario representing urban, mixed, and highway trip conditions. On daily basis, the implemented optimal solution proved the ability to reduce battery degradation and energy consumption by 15.8 % and 13 % respectively. The daily driving range has been accordingly extended by 8 % with minimal impact of 0.6 % on driveability. Moreover, an extended test has been conducted to yield the end-of-life of the battery after nearly 2900 days. On the long-term, the proposed method has been able to achieve up to 25 % extension of battery, preserving $<1$ % mitigation of driveability.

The proposed strategy in this work proved a significant potential at address multiple vital challenges of electric vehicles: battery degradation, energy/range anxiety, and driveability. The generic optimization problem, based on a high-fidelity driveline and battery degradation model, conduce to further investigation of design variables and customized objectives. Next steps of this work include real-time applicability of proposed strategy for limited time-horizons and the analysis of other features influencing the battery aging process.

## Data Availability

The datasets used and/or analyzed during the current study available from the corresponding author on reasonable request.
